# Bad Phages in Good Bacteria: Role of the Mysterious *orf63* of λ and Shiga Toxin-Converting Φ24_B_ Bacteriophages

**DOI:** 10.3389/fmicb.2017.01618

**Published:** 2017-08-25

**Authors:** Aleksandra Dydecka, Sylwia Bloch, Ali Rizvi, Shaili Perez, Bozena Nejman-Falenczyk, Gracja Topka, Tomasz Gasior, Agnieszka Necel, Grzegorz Wegrzyn, Logan W. Donaldson, Alicja Wegrzyn

**Affiliations:** ^1^Department of Molecular Biology, Faculty of Biology, University of Gdansk Gdansk, Poland; ^2^Department of Biology, York University Toronto, ON, Canada; ^3^Institute of Biochemistry and Biophysics, Polish Academy of Sciences Warsaw, Poland

**Keywords:** Shiga toxin-producing *Escherichia coli* (STEC), lambdoid bacteriophages, lytic development, *exo-xis* region, open reading frames

## Abstract

Lambdoid bacteriophages form a group of viruses that shares a common schema of genome organization and lifecycle. Some of them can play crucial roles in creating the pathogenic profiles of *Escherichia coli* strains. For example, Shiga toxin-producing *E. coli* (STEC) acquired *stx* genes, encoding Shiga toxins, via lambdoid prophages (Stx phages). The results obtained so far present the evidence for the relation between the *exo-xis* region of the phage genome and lambdoid phage development, however molecular mechanisms of activities of the *exo-xis* genes' products are still unknown. In view of this, we decided to determine the influence of the uncharacterized open reading frame *orf63* of the *exo-xis* region on lambdoid phages development using recombinant prophages, λ and Stx phage Φ24_B._ We have demonstrated that *orf63* codes for a folded protein, thus, it is a functional gene. NMR spectroscopy and analytical gel filtration were used to extend this observation further. From backbone chemical shifts, Orf63 is oligomeric in solution, likely a trimer and consistent with its small size (63 aa.), is comprised of two helices, likely intertwined to form the oligomer. We observed that the deletion of phage *orf63* does not impair the intracellular lambdoid phage lytic development, however delays the time and decreases the efficiency of prophage induction and in consequence results in increased survival of *E. coli* during phage lytic development. Additionally, the deletion of phage *orf63* negatively influences expression of the major phage genes and open reading frames from the *exo-xis* region during prophage induction with hydrogen peroxide. We conclude, that lambdoid phage *orf63* may have specific functions in the regulation of lambdoid phages development, especially at the stage of the lysis vs. lysogenization decision. Besides, *orf63* probably participates in the regulation of the level of expression of essential phage genes and open reading frames from the *exo-xis* region during prophage induction.

## Introduction

The significance of Shiga toxin-producing *E. coli* (STEC) as a public health problem was first recognized in 1982 during an investigation of an outbreak of hemorrhagic colitis associated with consumption of contaminated hamburgers (Riley et al., 1983). Since then, STEC strains have been implicated in many outbreaks of diarrhea world-wide. Quite recently (2011), the Shiga toxin-producing *E. coli* serotype O104:H4 was responsible for a serious epidemic outbreak in Germany (Bloch et al., 2012; Muniesa et al., 2012). STEC pathogens can cause serious food poisoning with bloody diarrhea in humans (Nataro and Kaper, 1998). Their main virulence factors are Shiga toxins, encoded by *stx* genes located in genomes of bacteriophages which occur in bacteria as prophages (Mizutani et al., 1999). These bacteriophages are called Shiga toxin-converting or Stx, for short, and belong to the lambdoid family of phages (Schmidt, 2001). All phages within this group indicate high similarities in the lifecycle and genomic organization to bacteriophage λ, the most reviewed member of this family (Wegrzyn et al., 2012). In the prophage state, most of phage genes, including *stx* genes, are not transcribed due to inhibition caused by the phage cI repressor. As a consequence, Shiga toxins are not produced under such conditions. Expression of *stx* as well as other phage genes occurs effectively only after prophage induction. In most cases, this process requires activation of the RecA-dependent bacterial S.O.S. response which is provoked by factors causing appearance of single-stranded DNA fragments. Activated RecA protein stimulates cleavage of the S.O.S. regulon repressor, the LexA protein, and the cI phage repressor. Prophage induction and subsequent phage lytic development lead to production of progeny phage particles and Shiga toxins, followed by their release from the lysed cell (Licznerska et al., 2016b). In the regulation of the lysis-vs. -lysogenization decision after infection of the host cell by a bacteriophage, both phage- and host-encoded proteins play important roles (for a review, see Wegrzyn et al., 2012). Among environmental factors influencing the decision, the crucial are temperature, nutrients availability and multiplicity of infection (m.o.i.). Lytic growth is supported by high temperature, rich medium and high m.o.i., while low temperature, starvation and low m.o.i. favor lysogenization. At the molecular level, the major players supporting lytic and lysogenic pathways are Cro and cI proteins, respectively. They are transcriptional regulators, and Cro represses expression of the *c*I gene, whereas cI downregulates transcription from two major “lytic” promoters (pL and pR, which provide mRNAs for *cro* and other “lytic” genes, encoding proteins involved in all processes during production of phage progeny) while stimulating its own expression by activation of the pM promoter. Thus, the result of the competition between Cro and cI is crucial for choosing one of the alternative developmental pathways. Since shortly after infection no cI protein is present, another transcription regulator, the cII protein (whose gene is transcribed from pR), is a key player in this game. This protein activates the second promoter for *c*I expression, pE. Therefore, cII activity decides on the Cro or cI predominance. In fact, cII is a subject of various regulatory mechanisms acting in response to different environmental conditions, including those playing major roles in the lysis-vs. -lysogenization decision (see Wegrzyn et al., 2012, for details).

**Figure 1:**
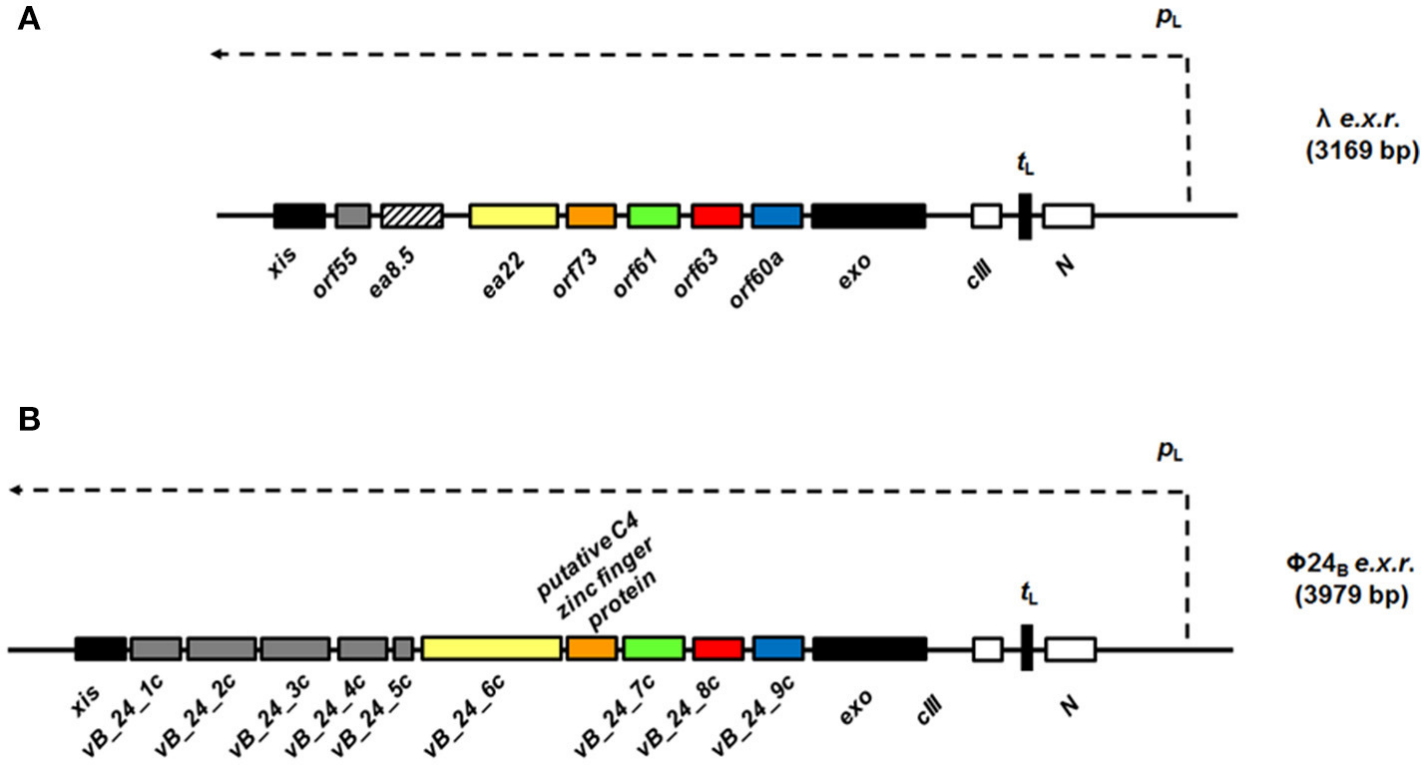
Maps of genes and open reading frames (ORFs) from the region located between *exo* and *xis* genes (black rectangles), the *exo-xis* region or *e.x.r.*, of lambdoid bacteriophages: λ **(A)** and Φ24_B_ **(B)**. In the case of phage λ **(A)**, the *exo-xis* region consists of two recognized genes: *ea22* and *ea8.5*, and five additional ORFs, named: *orf60a, orf63, orf61, orf73*, and *orf55*, which expression is under control of the *p*_L_ promoter (thin dashed arrow). Comparatively, the *exo-xis* region of phage Φ24_B_ **(B)** contains additional ORFs (gray rectangles), but there is no homolog of the *ea8.5* gene of phage λ **(A)**, rectangle with black stripes). Note, that some ORFs from the *exo-xis* region of phage Φ24_B_ **(B)**: *vb_24B_9c* (blue rectangles), *vb_24B_8c* (red rectangles), *vb_24B_7c* (green rectangles), *putative C4 zinc finger protein* (orange rectangles), and *vb_24B_6c* (yellow rectangles) are homologs of phage λ *orf60a, orf63, orf61, orf73, ea22* **(B)**, respectively. In spite of the differences in composition of both λ and Φ24_B_ *exo-xis* regions, attention needs to paid to highly conserved sequences of the *orf60a-orf73* regions among lambdoid bacteriophages (≥70% nucleotide and amino acid sequence identity) (Bloch et al., 2013). The regulatory genes: *N* and *c*III are marked as white rectangles and *t*_L_ terminator is indicated as black vertical rectangle.

An evolutionarily conserved region of lambdoid bacteriophage genome, located between *exo* and *xis* genes (so called “the *exo-xis* region”), contains several genes and open reading frames (Figure 1). Quite surprisingly, until recently, the role of this region in bacteriophage development was almost completely unknown. Recent studies indicated that overexpression of genes from the *exo-xis* region' impaired lysogenization of *E. coli* by bacteriophage λ (Loś et al., 2008b) and enhanced induction of prophages λ and Φ24_B_ (one of Shiga toxin-converting phages) (Bloch et al., 2013). The Ea8.5 protein, encoded by a gene located in the *exo*-xis region, contains a fused homeodomain/zinc-finger fold (Kwan et al., 2013), which suggest a regulatory role for this protein. Interestingly, prophage induction with mitomycin C or hydrogen peroxide caused different expression patterns of genes from the *exo-xis* region; such differences were observed in both phages, λ and Φ24_B_ (Bloch et al., 2014). Moreover, phages with deletions in the *exo-xis* region responded to the oxidative stress in a different manner relative to wild-type phages (Licznerska et al., 2016a). Therefore, it is important to determine structures and functions of particular proteins encoded in the *exo-xis* region.

In this work, we have focused on *orf63*. In our preliminary experiments with mutants in particular genes and ORFs from the *exo-xis* region, deletion of *orf63* gave one of the strongest effects (Licznerska et al., 2016a). Moreover, the transcription factor YqhC has been recognized as a potential partner for interaction with Orf63 in yeast two-hybrid study of phage-host interactions (Blasche et al., 2013). Therefore, we decided to investigate structure and functions of *orf63* and its product, the Ofr63 protein, in more detail.

## Materials and methods

### The *orf63* gene expression and protein purification

A codon-optimized *orf63* gene (NCBI ID: 2703507) for high level expression *E. coli* was synthesized by ATUM (Menlo Park, CA) and supplied in plasmid pD441-NH for direct transformation of a BL21 host strain (Novagen). Amino terminal 6xHis and Flag (DYKDDDDK) tags were included to facilitate affinity purification and detection. Milligram quantities of isotopically labeled 6xHis-Flag-Orf63 for NMR spectroscopy were obtained from a 1.0 L fermentation in a minimal medium containing 1 g ^15^NH_4_Cl, 3 g of ^13^C-glucose, and 1 g of ^15^N-^13^C Celtone algal extract (CIL; Cambridge, MA). The cell pellet was dissolved in T300 buffer (20 mM Tris-HCl, 300 mM NaCl, 0.05% NaN_3_) and lysed by French press and sonication. The Orf63 protein purified from the bacterial soluble fraction by Nickel-NTA affinity chromatography (Qiagen) that included a 10 mM imidazole wash step and a 20 mM EDTA elution step, all in T300 buffer. A subsequent gel filtration chromatography step (Sephacryl-100, HiLoad 16/60; GE Life Sciences) was employed to further purify the Orf63 protein and exchange it into NMR spectroscopy buffer (5 mM Tris-HCl, 0.15 M NaCl, 0.05% NaN_3_).

### Gel filtration assay to estimate apparent molecular weight

Chromatograms of eleven proteins on the same gel filtration column used to purify Orf63 were compiled to produce a standard curve describing the relationship between retention volume and molecular weight. Specific proteins used for the standard curve included the HACS1 SH3 domain (10.6 kDa), the AIDA1 PTB domain (22.0 kDa), the monomeric and dimeric states CASKIN2 SAM domain tandem (20.2 / 40.5 kDa), the CASKIN2 SAM1 domain (10.3 kDa), the SHP2 adaptor SH2 domain (14.1 kDa), the AIDA1 SAM domain tandem (16.4 kDa), two deletion mutants of the La RRM domain (14.6 / 15.9 kDa), the Crk2 adaptor SH2 domain (15.5 kDa), dimeric glutathione S-transferase (52 kDa), and calmodulin (18.8 kDa).

### NMR spectroscopy

A 0.5 mM sample of ^13^C,^15^N-labeled Orf63 was prepared for NMR spectroscopy in NMR buffer supplemented with 10% D_2_O. All experiments were performed at 310 K using a Bruker Avance 700 MHz NMR spectrometer equipped with a cryogenically cooled 5 mm probe at the York University Life Sciences Building Central Facility. Backbone (HN, N, CA, CB, C') assignments were achieved using a set of conventional triple resonance experiments (HNCA, HNCACB, CBCAcoNH, HNCO, HNcaCO) incorporating sparse sampling for the optimum sensitivity and resolution. Datasets were processed with NMRpipe (Delaglio et al., 1995) and istHMS (Hyberts et al., 2012) and interpreted with CCPN Analysis (Skinner et al., 2015).

### Bacteria, bacteriophages and plasmids

The *E. coli* strains, bacteriophages and plasmids used in *in vivo* work are presented in Table 1. Work with these strains was approved by the Ministry of Environment (decision no. 189/2016). The *E. coli* lysogens were obtained using the following lambdoid phages: λ, λΔ*orf63*, Φ24_B_ or Φ24_B_Δ*orf63* (Bloch et al., 2013; Licznerska et al., 2016b). In the first step, phage lysates were prepared. Bacterial cultures were grown at 37°C to A_600_ = 0.1. Then, mitomycin C (Sigma—Aldrich) was added to all flasks to a final concentration of 1 μg/ml. The incubation with shaking was continued for about 12 h. To obtain lysates, bacterial debris were centrifuged (2,000 × g for 10 min at 4°C) and supernatants were filtered through the 0.22-μm-pore-size filters (Sigma—Aldrich). In the next step, the lysogenization procedure was carried out. Briefly, *E. coli* strain C600 was cultivated at 37°C to A_600_ = 0.2. Then, 4 ml of bacterial culture was centrifuged (2,000 × g for 5 min at RT), the pellet was washed with TCM buffer (10 mM Tris-HCl, 10 mM MgSO_4_, 10 mM CaCl_2_, pH 7.2; Sigma—Aldrich) and suspended in LB medium (Sigma—Aldrich) supplemented with MgSO_4_ (phages λ and λΔ*orf63*) or with MgSO_4_ and CaCl_2_ (phages Φ24_B_ and Φ24_B_Δ*orf63*) to a final concentration of 10 mM. Bacteriophages were added to the suspensions to m.o.i. of 10. Following incubation at 37°C, the mixtures were spread on LB agar plates. After overnight incubation at 37°C, bacterial colonies were tested for the presence of prophages by using UV irradiation (this procedure is described in detail in the next section). For construction of the plasmid pSB_orf63_λ, nucleotide sequence of *orf63* from phage λ was amplified by PCR with primers: Fλorf63_EcoRI (5′GGA GAA TTC GGC TGT ATG CAC AAA GC) and Rλorf63_BamHI (5′ GAG GAT CCT GCA TTC CGT GGT TGT C), and phage DNA as a template, which was isolated by using MasterPure™ Complete DNA and RNA Purification Kit (Epicenter). Then, the λ*orf63* was ligated with fragment of plasmid pUC18 (insert and vector were digested with EcoRI and BamHI restrictions endonucleases; Thermo Scientific), bearing an ampicillin resistance gene and sequence of *p*_lac_ promoter. The plasmid pSB_orf63_Φ24_B_ was constructed according to similar procedure. To amplify a DNA fragment containing *vb_24B_8c* sequence (the homolog of λ*orf63*) by PCR method, two primers: FΦ24_B_orf63_EcoRI (5′GGA GAA TTC GGC TGT ATG CAC AAA GC) and RΦ24_B_orf63_BamHI (5′GTA GGA TCC TTG TCA TGC CGG GTC) were used. Next, plasmid pUC18 and insert were cut with EcoRI and BamHI enzymes and ligate by the T4 DNA ligase (Thermo Scientific). The construction of pUC18 derivatives was confirmed by DNA sequencing (Genomed).

**Table 1:** Bacterial strains, bacteriophages and plasmids used for *in vivo* experiments.

***E. coli* strains, bacteriophages or plasmids**	**Relevant genotype or description**	**References**
***E. coli*** **LABORATORY STRAINS**		
MG1655	F– λ– *ilvG rfb-50 rph-1*	Jensen, 1993
MG1655 (λ)	MG1655 bearing λ prophage	Bloch et al., 2013
MG1655 (λΔ*orf63*)	MG1655 bearing λ prophage with deletion of *orf63*	Licznerska et al., 2016a
MG1655 (Φ24_B_)	MG1655 bearing Φ24_B_ prophage	Bloch et al., 2013
MG1655 (Φ24_B_Δ*orf63*)	MG1655 bearing Φ24_B_ prophage with deletion of *vb_24B_8c*, the homolog of λ*orf63*	Licznerska et al., 2016a
C600	F– *tonA21 thi-1 thr-1 leuB6 lacY1 glnV44 rfbC1 fhuA1 λ^−^*	Appleyard, 1954
C600 (λ)	C600 bearing λ prophage	This study, by lysogenization
C600 (λΔ*orf63*)	C600 bearing λ prophage with deletion of *orf63*	This study, by lysogenization
C600 (Φ24_B_)	C600 bearing Φ24_B_ prophage	This study, by lysogenization
C600 (Φ24_B_Δ*orf63*)	C600 bearing Φ24_B_ prophage with deletion of *vb_24B_8c*, the homolog of λ*orf63*	This study, by lysogenization
**BACTERIOPHAGES**		
λ	carries a frameshift mutation relative to Ur-lambda	Hendrix and Duda, 1992
λΔ*orf63*	Φ24_B_ phage with deletion of *orf63*	Licznerska et al., 2016
Φ24_B_	λs*tx2*::*cat*	Allison, 2003
Φ24_B_Δ*orf63*	Φ24_B_ phage with deletion of *vb_24B_8c*, the homolog of λ*orf63*	Licznerska et al., 2016
**PLASMIDS**		
pUC18	*ori* pMB1 (pBR322 derivative), *bla*, Amp^R^	Thermo fisher scientific
pSB_orf63_λ	as pUC18 but bearing the *orf63* from phage λ	This study
pSB_orf63_Φ24_B_	as pUC18 but bearing the *orf63* from phage Φ24_B_	This study

### Media and growth conditions

All *in vivo* experiments were performed in LB liquid medium (Sigma—Aldrich) supplemented with 10 mM MgSO_4_ (phage λ or phage λΔ*orf63*) or with 10 mM MgSO_4_ and 10 mM CaCl_2_ (phage Φ24_B_ or phage Φ24_B_Δ*orf63*), and with 50 μg/ml ampicillin (if necessary) (Sigma—Aldrich). To stimulate Orf63 protein production from the recombinant pUC18 derivatives, overnight bacterial cultures were diluted 1:100 in fresh LB medium and treated with IPTG (A&A Biotechnology) to a final concentration of 1 mM. Then, host bacteria were grown in aeration condition, achieved by shaking, at 30°C to A_600_ = 0.1 or 0.2 (the optical density of bacterial cells was dependent on the experimental conditions described in the following chapters).

### Double overlay plaque assay

Bacteriophage titration was performed on the standard Petri dishes (Alchem) filled with 25 ml of LB agar (1.5% agar; Sigma—Aldrich), according to a procedure described by Sambrook and Russell (2001), with some modification. The top layer was prepared by mixing 2 ml of LB agar (0.7% agar; Sigma—Aldrich) with 1 ml of the overnight bacterial cell culture. To obtain visible plaques formed by Stx phages, the bottom agar was supplemented with sublethal concentration of chloramphenicol (Sigma—Aldrich). This antibiotic was effective in increasing of size of plaques of phage Φ24_B_ and its derivative, which possessed in genomes chloramphenicol resistance gene (Table 1). As described previously (Loś et al., 2008a), the *cm* gene expression, especially after phage infection of *E. coli* bacteria, may have the positive influence on cellular productivity by decreasing the inhibitory effects of the antibiotic on protein synthesis. To determine the number of phages per ml of suspension (PFU/ml), serial 10-fold dilutions were prepared in TM buffer (10 mM Tris–HCl, 10 mM MgSO_4_; pH 7.2). Then, appropriate volume of each dilution of phage lysate was spotted onto double agar layer. The plates were incubated at 37°C overnight, plaques were counted, and the phage titer was calculated.

### One-step growth experiments in phage-infected bacteria

To investigate the intracellular lytic development of lambdoid phages the one-step-growth experiment was prepared using the method described by Wegrzyn et al. (1995), with a minor modification (Bloch et al., 2014; Nejman-Falenczyk et al., 2015). Host bacteria were grown in LB medium at 30°C to A_600_ = 0.2. In the next step, 10 ml of a bacterial culture was centrifuged (2,000 × g for 10 min at 4°C). The pellet was suspended in 1 ml of LB medium supplemented with 3 mM sodium azide (Sigma—Aldrich). Bacteriophages were added to *E. coli* cells to m.o.i. of 0.05. After 10 min incubation at 30°C, unadsorbed phages were removed by three times washing in LB medium with 3 mM sodium azide (2,000 × g for 10 min at 4°C). Then, 25 μl of the suspension was added to 25 ml of LB medium prewarmed to 30°C (time 0) and cultivated in an incubator shaker. The number of infection centers were determined at times: 5, 10, 15 min after infection by mixing 0.1 ml of the sample with 0.9 ml of an overnight culture of appropriate indicator bacteria and 2 ml of top agar. Next, the mixture was poured onto LB agar plate (phages λ and λΔ*orf63*) or LB agar plate with 2.5 μg/ml chloramphenicol (phage Φ24_B_ and Φ24_B_Δ*orf63*). Samples taken at later times were treated with chloroform (POCH), shaken vigorously and cleared by centrifugation (2,000 × g for 5 min at RT). The phage lysate was diluted in TM buffer and titrated under permissive condition. Plates were incubated at 37°C overnight. The number of viruses released from each infected cell (burst size) was calculated as a ratio of phage titer to the titer of infection centers.

### Prophage induction with hydrogen peroxide

Bacteria lysogenic for lambdoid phages were grown in LB medium at 30°C to A_600_ = 0.1. Next, the culture was divided into two aliquots. One of them was treated with 1 mM hydrogen peroxide (Sigma—Aldrich) to provoke the prophage induction. The second one was a control without an induction agent. The cultivation was continued at 30°C. At indicated times samples were harvested, mixed with chloroform and vortexed for 1 min. The suspension was centrifuged for 5 min in a microfuge at RT. The supernatant was diluted in TM buffer and 2.5 μl of each serial dilution was dropped onto a freshly prepared double-layer LB agar in plastic Petri dishes. Plates were incubated at 37°C overnight. The relative phage titer was estimated by subtracting the phage titer determined in non-induced cultures from the phage titer estimated in induced cultures.

### Survival of host bacteria after bacteriophage infection

To estimate the percentage of surviving cells after bacteriophage infection the procedure created by Sambrook and Russell (2001) was used, with a minor modification. A bacterial culture was grown at 30°C to A_600_ = 0.2. Then, 4 ml of the sample was centrifuged (2,000 × g for 10 min at 4°C). The supernatant was discarded and the pellet was washed with 0.85% sodium chloride (POCH) (2,000 × g for 10 min at 4°C). Finally, the bacterial pellet was suspended in 1 ml of LB medium supplemented with MgSO_4_ (phage λ and phage λΔ*orf63*) or with MgSO_4_ and CaCl_2_ (phage Φ24_B_ and Φ24_B_Δ*orf63*) to a final concentration of 10 mM. The suspension was incubated for 30 min at 30°C and then phage particles were added to m.o.i. of 1, 5, 10. The mixture was kept for 15 min (phage λ and phage λΔ*orf63*) or 30 min (phage Φ24_B_ and Φ24_B_Δ*orf63*) at 30°C. In the next step, serial dilutions in 0.85% sodium chloride were prepared and 40 μl of each dilution was spread on LB agar plates. After overnight incubation at 37°C, percentage of surviving *E. coli* bacteria was calculated relative to bacterial culture in which TM buffer was added instead of phage particles.

### Efficiency of prophage formation after bacterial virus infection

Efficiency of lysogenization was estimated according to Arber et al. (1983) and Wegrzyn et al. (1992), with some modification. Host bacteria were cultured at 30°C to A_600_ = 0.2. Next, 1 ml of the sample was centrifuged (2,000 × g for 10 min at 4°C). Bacterial culture was washed with TCM buffer twice, and then pellet was suspended in the same buffer. Bacteriophages were added to bacterial cells to m.o.i. of 1, 5, 10. The mixture was incubated at 30°C. Then, serial dilutions were prepared and 20 μl of each suspension was spread on LB agar plates prior to overnight incubation at 37°C. The next day, 96 colonies were passaged in each well of a 96-well plate with 200 μl of LB medium and shaken at 37°C to A_600_ = 0.1. To estimate a percent of lysogens among survivors, bacterial cultures were treated with UV light at 50 J/m^2^ (the dose used routinely for lambdoid prophage induction) and incubated at 37°C for 2 h. Following induction, putative lysogens were mixed with chloroform, centrifuged (2,000 × g for 10 min at 4°C) and the water phase was spotted onto a double-layer LB agar (phage λ and phage λΔ*orf63*) or a double-layer LB agar supplemented with chloramphenicol to a final concentration of 2.5 μg/ml (phage Φ24_B_ and Φ24_B_Δ*orf63*). Efficiency of lysogenization was calculated as a percent of lysogens relative to all tested bacterial cells. Lysogens were also infected with the same phage to check their resistance to superinfection, as described previously (Wegrzyn et al., 1992).

### Prophage induction and extraction of RNA

Induction of tested prophages was provoked in lysogenic bacteria by addition of hydrogen peroxide to a final concentration of 1 mM. At the appropriate time, 10^9^ bacterial cells were harvested, treated with 10 mM sodium azide and deep frozen in liquid nitrogen (this procedure was necessary to inhibit the growth of host bacteria). Total RNA from all samples were isolated with the High Pure RNA Isolation Kit (Roche Applied Science). To remove DNA from RNA preparations the TURBO DNA-free™ Kit (Life Technologies) was used. The quality and quantity of total isolated RNA were analyzed by a NanoDrop spectrophotometer and agarose gel electrophoresis. The contamination of DNA from RNA samples was also tested by routine PCR and qRT-PCR.

### cDNA synthesis from an RNA template

To synthesize cDNA from an RNA template, the Transcriptor Reverse Transcriptase and random hexamer primers (Roche Applied Science) were used, according to the protocol supplied from the provider. 1.25 μg of the total RNA was taken for each reaction. Finally, mixture was diluted 10-fold and tested in qRT-PCR.

### qRT-PCR assay and data analysis

The pattern of genes expression after prophage induction was performed by using the LightCycler® 480 Real-Time PCR System (Roche Applied Science), LightCycler® 480 SYBR Green I Master (Roche Applied Science) and cDNA samples. Transcription rates of genes of lambdoid bacteriophages were compared in parallel to the *16S rRNA* housekeeping gene (according to a procedure described by Strauch et al. (2008), which expression was stable during prophage induction provoked by hydrogen peroxide. All primers were created by Primer3web version 4.0.0 and are listed in Table 2. Each reaction mixture consisted of: 2x SYBR Green I Master Mix, 6.25 ng/μl cDNA and 200 nM specific primers. qRT-PCR amplifications were performed for 55 cycles. To confirm the specificity of primers, melting curve for each product was analyzed. The relative changes in gene expressions were determined by E-Method and calculated by the following formula: Normalized relative ratio = E_t_^CT (target) calibrator−CT (target) sample^ / E_r_^CT (reference) calibrator−CT (reference) sample^, where E_t_ is the PCR efficiency of target and E_r_ means the PCR efficiency of reference. The sample before the addition of the inductor (the time point “zero”) was a calibrator. The raw run data for tested lambdoid phages were transferred using the “LC480 Conversion: conversion of raw LC480 data” software and then, PCR efficiency for each gene was calculated by LinRegPCR program, which was successfully used previously (Bloch et al., 2014, 2015; Nejman-Falenczyk et al., 2015; Licznerska et al., 2016a).

**Table 2:** Primers used for RT-Qpcr.

**Primer name**	**Sequence (5′ → 3′)**
pF_λ_ea8.5	GGGCAAGTATCGTTTCCACC
pR_ λ_ea8.5	GCAATGTGCGAGAAATGACTG
pF_λ_ea22	GCAGTTCCAGCACAATCGAT
pR_ λ_ea22	AATGCATGACGACTGGGGAT
pF_λ_orf73	CACTTCGAACCTCTCTGTTTACTG
pR_ λ_orf73	CAGGGTTGTCGGACTTGTG
pF_λ_orf61	TTAGCCTGACGGGCAATG
pR_ λ_orf61	CCGACATGGGACTTGTTCA
pF_λ_orf60a	GCATACAGCCCCTCGTTTAT
pR_ λ_orf60a	CCGAAATCCACTGAAAGCAC
pF_λ_cIII	ATTCTTTGGGACTCCTGGCTG
pR_ λ_cIII	GTAAATTACGTGACGGATGGAAAC
pF_λ_N	CTCGTGATTTCGGTTTGCGA
pR_ λ_N	AAGCAGCAAATCCCCTGTTG
pF_λ_cro	ATGCGGAAGAGGTAAAGCCC
pR_ λ_cro	TGGAATGTGTAAGAGCGGGG
pF_λ_cII	TCGCAATGCTTGGAACTGAGA
pR_ λ_cII	CCCTCTTCCACCTGCTGATC
pF_λ_Q	TTCTGCGGTAAGCACGAAC
pR_ λ_Q	TGCATCAGATAGTTGATAGCCTTT
pF_λ_R	ATCGACCGTTGCAGCAATA
pR_ λ_R	GCTCGAACTGACCATAACCAG
pF_Φ24B_ea22	TCAGCAACATGGCATTCACT
pR_ Φ24B_ea22	GGTTGGGAAGCTGAGAGTTG
pF_Φ24B_orf73	CGAACCTCTCTGTTTACTGATAAGC
pR_ Φ24B_orf73	TTCAGGGTTGTCGGACTTGT
pF_Φ24B_orf61	TTAGCCTGACGGGCAATG
pR_ Φ24B_orf61	CCGACATGGGACTTGTTCA
pF_Φ24B_orf60a	CATACAGCCCCTCGTTTAT
pR_ Φ24B_orf60a	CCGAAATCCACTGAAAGCAC
pF_Φ24B_cIII	ATTCTTTGGGACTCCTGGCTG
pR_Φ24B_cIII	GTAAATTACGTGACGGATGGAAAC
pF_Φ24B_N	AGGCGTTTCGTGAGTACCTT
pR_ Φ24B_N	TTACACCGCCCTACTCTAAGC
pF_Φ24B_cro	CGAAGGCTTGTGGAGTTAGC
pR_ Φ24B_cro	GTCTTAGGGAGGAAGCCGTT
pF_Φ24B_cII	TGATCGCGCAGAAACTGATTTAC
pR_Φ24B_cII	GACAGCCAATCATCTTTGCCA
pF_Φ24B_O	AAGCGAGTTTGCCACGAT
pR_Φ24B_O	GAACCCGAACTGCTTACCG
pF_Φ24B_Q	GGGAGTGAGGCTTGAGATGG
pR_ Φ24B_Q	TACAGAGGTTCTCCCTCCCG
pF_Φ24B_R	GGGTGGATGGTAAGCCTGT
pR_ Φ24B_R	TAACCCGGTCGCATTTTTC
pF_E.coli_16SrRNA	CCTTACGACCAGGGCTACAC
pR_ E.coli_16SrRNA	TTATGAGGTCCGCTTGCTCTC

### Statistical analysis

Each experiment was repeated three times and variation among replicates was presented as the error bars indicating the standard deviation (SD). All data comparisons were made by using Student's *t*-test. Significant differences were marked by asterisks when *P* < 0.05 (^*^) or *P* < 0.01 (^**^).

## Results

### The oligomeric state of Orf63

**Figure 2:**
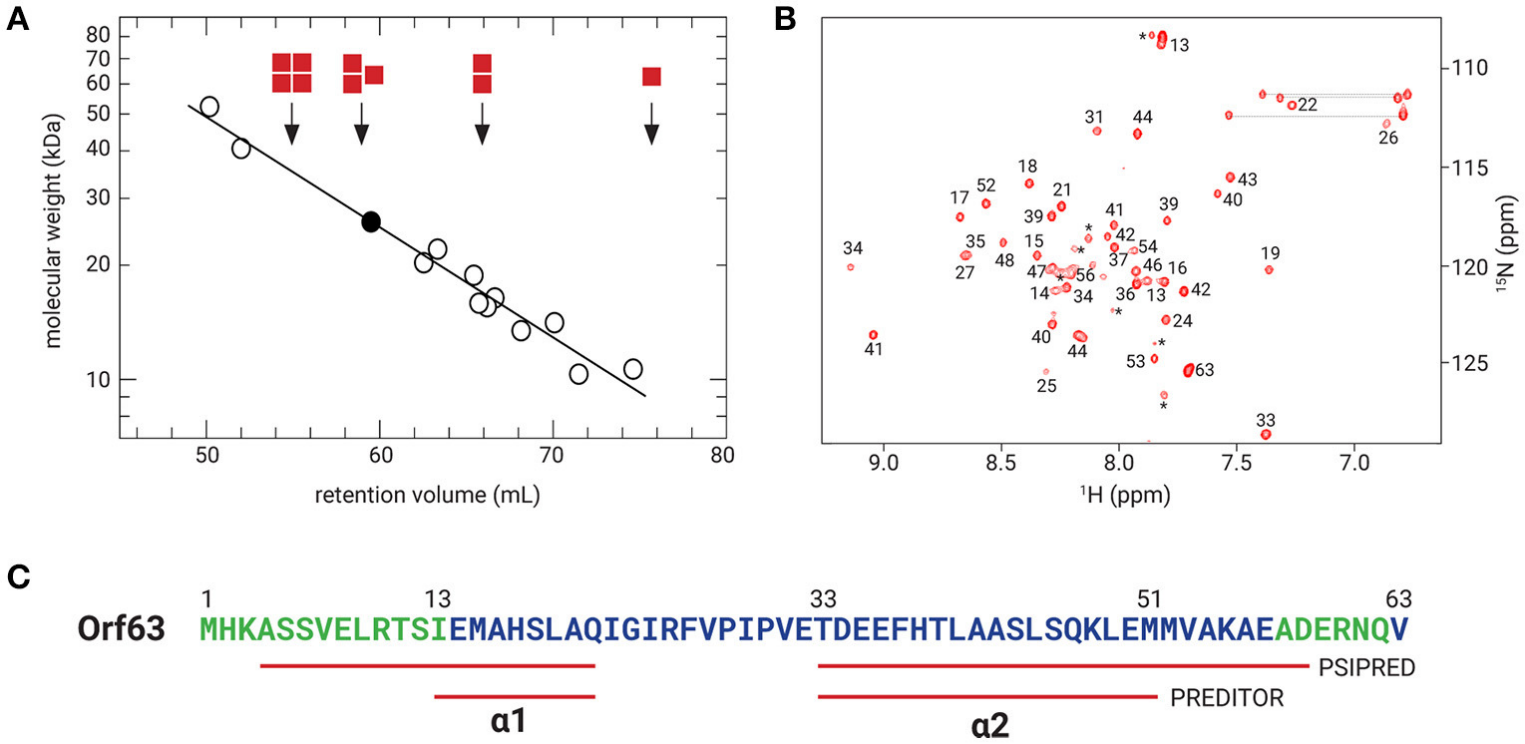
Orf63 structural insights. **(A)** The retention volumes for a set of proteins ranging from 10–67 kDa were plotted against molecular weight to produce the log-linear relationship shown (open circles). The K_av_ value observed for Orf63 corresponds to an apparent molecular weight of ~28 kDa (closed circle). For reference, red squares denote the expected retention volumes of monomeric, dimeric, trimeric, and tetrameric species of the 9 kDa affinity tagged Orf63 protein. **(B)** A representative ^1^H-^15^N HSQC spectrum of Orf63 at 37°C demonstrating the characteristic resonance dispersion of a folded protein. Unassigned residues are labeled with an asterisk. Lines denote the amino resonances of three asparagine and glutamine side chains. **(C)** A conventional strategy was used to assign the backbone (HN, N, CA, CB, C') chemical shifts of Orf63. The extent of these assignments is colored blue on the sequence. The chemical shift data were then used as input to PREDITOR (Berjanskii et al., 2006) which identified two helices labeled α1 and α2. Using sequence data alone as input to PSIPRED (McGuffin et al., 2000), longer helices are predicted.

Samples from four independent preparations of 6xHis-Flag tagged Orf63 (15 aa. tag + 63 aa. protein) eluted as one peak on a preparative gel filtration column with an average retention volume of 59.5 mL corresponding to an apparent molecular weight of ~26 kDa (Figure 2A). Since affinity-tagged Orf63 is only 9 kDa, the gel filtration results suggest that Orf63 is oligomeric with a trimer as the most plausible configuration. This estimate is most accurate if Orf63 has the characteristics of a globular protein to match the standards used.

### The *orf63* gene encodes a folded protein

Consistent with the observation that Orf63 is oligomeric in solution, NMR spectra of Orf63 at 298 K (25°C) suffered from considerable resonance line broadening that was characteristic of proteins >20 kDa in overall molecular weight. Consequently, a higher temperature of 310 K (37°C) was chosen for all NMR studies to increase the tumbling time of the protein that, in turn, improves the sensitivity of triple resonance experiments. In Figure 2B, a ^1^H-^15^N HSQC spectrum is presented. The amide resonances in this two-dimensional spectrum are disperse indicating that the protein is folded. The combined analysis of several triple resonance (^1^H, ^13^C, ^15^N) spectra lead to the determination of backbone (HN, N, CA, CB, C') chemical shift assignments for residues 14–52 of Orf63. Resonances for the amino terminal affinity tags and from residue 53 onwards to the carboxy-terminus were either not observed or unassignable. Thus, the NMR data suggest that the folded region of Orf63 includes from residues 14–52.

### Structural characteristics of Orf63

Several statistical methods are available to predict secondary structure from backbone chemical shift data with a high degree of accuracy. As shown in Figure 2C, two helices are predicted (α1: 13–21; α2: 33–50). The secondary structure determined from chemical shift data is consistent with the secondary structure of Orf63 predicted from sequence information alone, although the helical boundaries are different.

### The sequence of putative *orf63* products is conserved among lambdoid bacteriophages

Since experiments shown in Figure 2 indicated that *orf63* encodes a protein, we have tested similarity of the putative proteins encoded by *orf63* of different lambdoid bacteriophages. Thus, scores of pairwise alignments of the predicted amino acid sequences of *orf63* from six such phages have been calculated. As demonstrated in Table 3, all these putative proteins are similar to each other. This indicate that the high similarity is kept at the protein level of Orf63 of lambdoid bacteriophages.

**Table 3:** Scores of pairwise alignments of the predicted amino acid sequences of *orf63* from six analyzed lambdoid phages: λ phage (NC_001416), Φ24_B_ phage (HM208303), 933W phage (NC_000924), VT2 Sakai phage (AP000422), Stx1 converting phage (NC_004913), and Stx2 converting phage II (NC_004914).

	**λ**	**Φ24_B_**	**933W**	**VT2 Sakai**	**Stx1**	**Stx2_II**
λ		86	86	87	87	87
Φ24_B_			100	98	98	98
933W				98	98	98
VT2 Sakai					100	100
Stx1						100
Stx2_II						

*Pairwise scores are simply the number of identities between the two sequences, divided by the length of the alignment, and represented as a percentage. The multiple sequence alignment was performed using the ClustalW algorithm*.

### Efficiency of lysogenization and prophage induction in the absence of *orf63*

**Figure 3:**
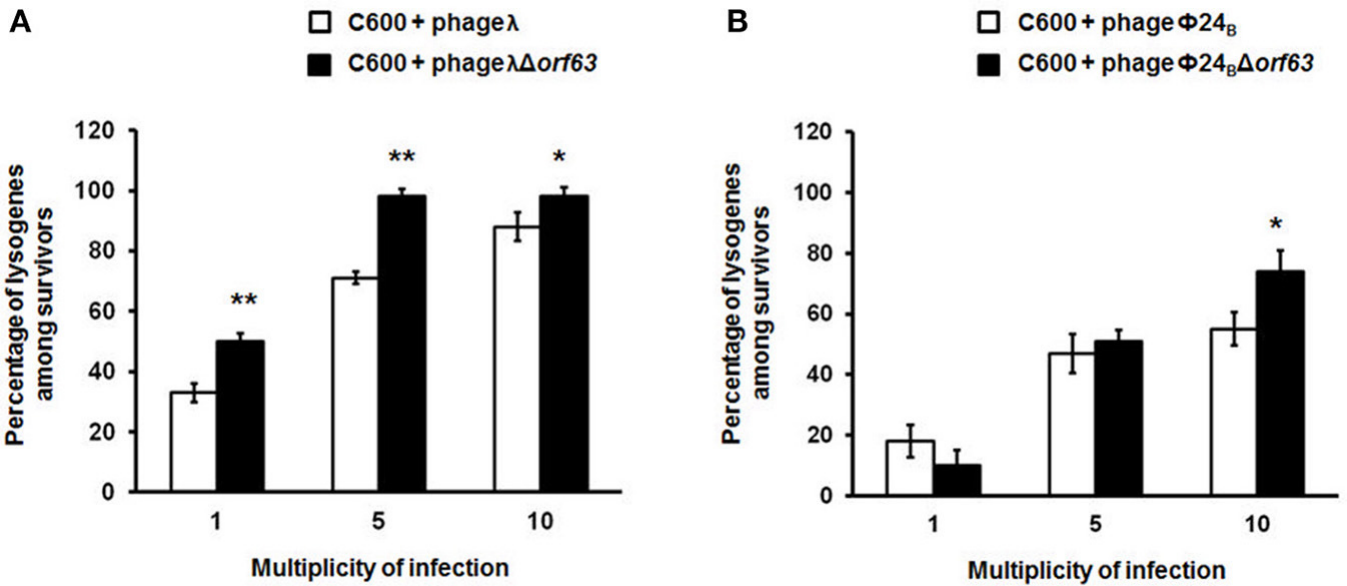
Efficiency of lysogenization of *E. coli* C600 strain with lambdoid bacteriophages: λ and Φ24_B_ (□ in **(A,B)**, respectively) or their deletion mutants λΔ*orf63* and Φ24_B_Δ*orf63* (■ in **A,B**, respectively). Results are presented as mean values ±*SD* from three independent experiments. Statistical analysis (*t* test) was performed for results from each m.o.i. (multiplicity of infection) between wild type phage and its deletion mutant. Significant differences are marked by asterisks *P* < 0.05 (^*^) or *P* < 0.01 (^**^).

**Figure 4:**
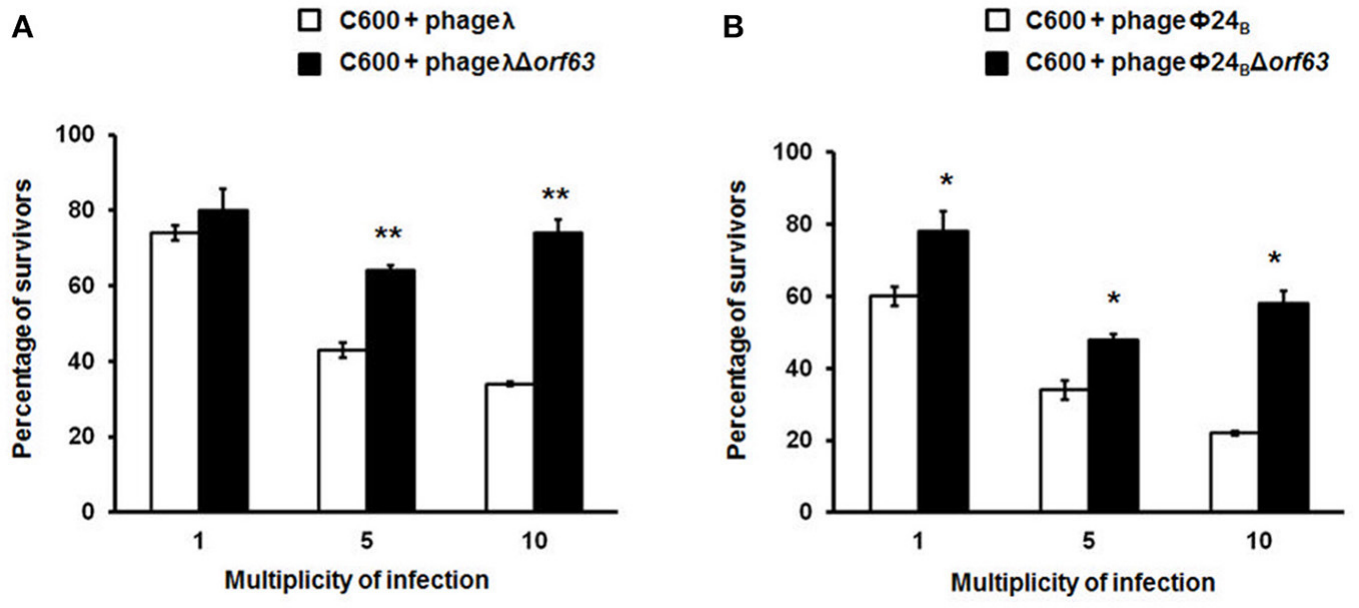
Survival (%) of the wild-type strain *E. coli* C600, after infection with lambdoid bacteriophages: λ and Φ24_B_ (□ in **(A,B)**, respectively) or their deletion mutants λΔ*orf63* and Φ24_B_Δ*orf63* (■ in **A,B**, respectively). Mean values from three independent experiments ±*SD* are shown. Statistical analysis were performed for each m.o.i. by *t* test. The significance of differences between fractions of bacterial cells surviving the infection with λ and λΔ*orf63* as well as Φ24B and Φ24BΔ*orf63* are observed and marked by asterisks *P* < 0.05 (^*^) or *P* < 0.01 (^**^).

Since previous studies suggested that genes from the *exo-xis* region might be involved in the regulation of bacteriophage development (Bloch et al., 2013, 2014; Licznerska et al., 2016a), we have tested two crucial controlled steps in the lambdoid phage life cycle, the lysis-vs.-lysogenization decision, and prophage induction. We found that lysogenization efficiency was significantly increased in bacteriophages λ and Φ24_B_ devoid of *orf63* (Figures 3A,B, respectively) though this phenomenon was more pronounced in λ (the effects were seen at all tested m.o.i.) (Figure 3A) than in Φ24_B_ (significant effects observed only at m.o.i. = 10) (Figure 3B). Also, survival rates of bacterial cells (i.e., cells lysogenized and not infected) in populations infected with Δ*orf63* or Φ24_B_ Δ*orf63* were higher than those in experiments with wild-type λ or Φ24_B_ (Figures 4A,B, respectively), supporting the conclusion that lysogenization is more effective for the mutant, indeed.

**Figure 5:**
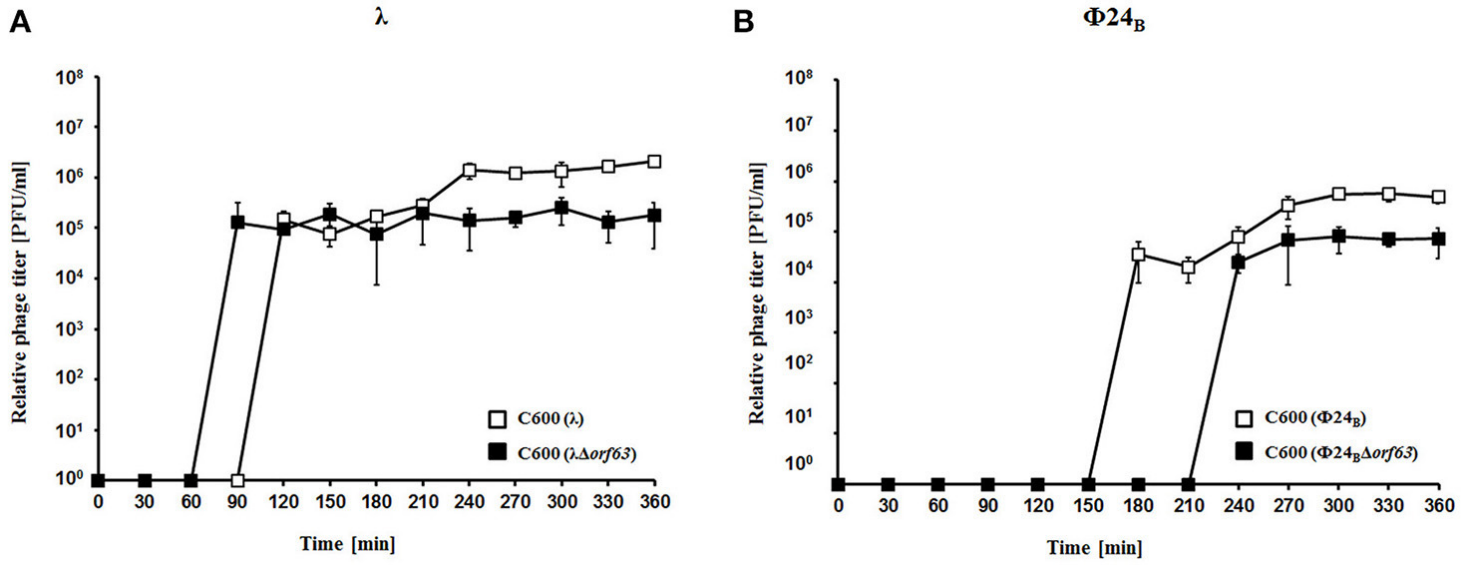
Development of λ and λΔ*orf63* **(A)** or Φ24_B_ and Φ24_B_Δ*orf63* **(B)** bacteriophages after prophage induction with hydrogen peroxide at 30 °C. *E. coli* C600 bacteria were either lysogenic with wild-type phages λ and Φ24_B_ (□ in **A,B**, respectively) or their deletion mutants λΔ*orf63* and Φ24_B_Δ*orf63* (■ in (**A,B**, respectively). Phage lytic development was initiated by addition of H_2_O_2_ to final concentration of 1 mM at time 0. The presented results are mean values from three independent experiments with error bars indicating *SD* (note that in the most cases, the bars are smaller than sizes of symbols). Results are shown as PFU (plaque forming units) per one ml.

**Figure 6:**
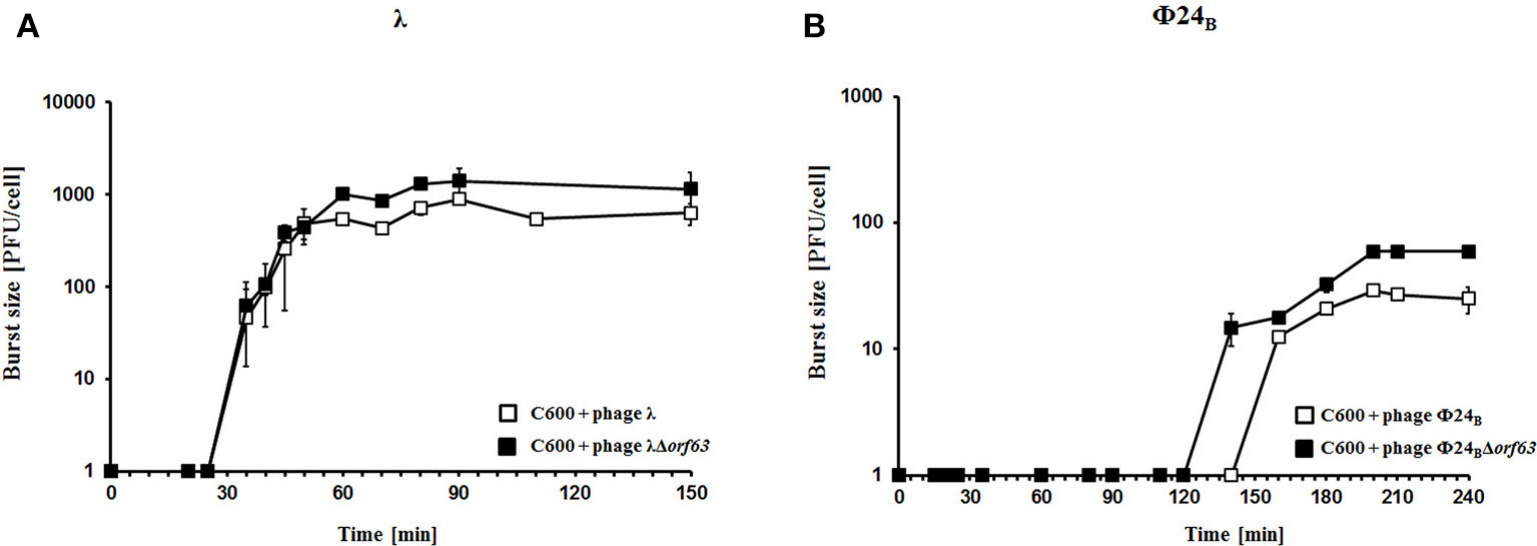
Development of λ and λΔ*orf63* **(A)** or Φ24_B_ and Φ24_B_Δ*orf63* **(B)** bacteriophages following phage infection of *E. coli* bacteria. Host *E. coli* strains were infected with wild-type phages λ and Φ24_B_ (□ in **A,B**, respectively) or their deletion mutants λΔ*orf63* and Φ24_B_Δ*orf63* (■ in **A,B**, respectively) at time 0. The presented results are mean values ±*SD* from three independent experiments. Results are shown as PFU (plaque forming units) per cell.

To test efficiency of prophage induction, we have estimated the number of phages appearing after prophage induction with hydrogen peroxide (one of natural prophage inducers occurring in human intestine, the common habitat of *E. coli*). Deletion of *orf63* caused a lower phage titer after prophage induction for both λ and Φ24_B_ (Figures 5A,B, respectively). However, when measured kinetics of phage development following infection of *E. coli* cells at low m.o.i. (0.05), we found that phages λ and Φ24_B_ devoid of *orf63* gave even more progeny per infected cell than their wild-type counterparts (Figures 6A,B, respectively). Therefore, combining results of experiments presented in Figures 5, 6, one can conclude that deletion of *orf63* influences efficiency of prophage induction in both λ and Φ24_B_. Since formation of progeny phages is definitely not impaired in the absence of *orf63* when lytic development starts after infection, we suggest that lower phage titer after prophage induction indicates lower efficiency of this process in phages devoid of this gene.

### Deletion of *Orf63* influences expression of genes of λ and Φ24_B_ phages

**Figure 7:**
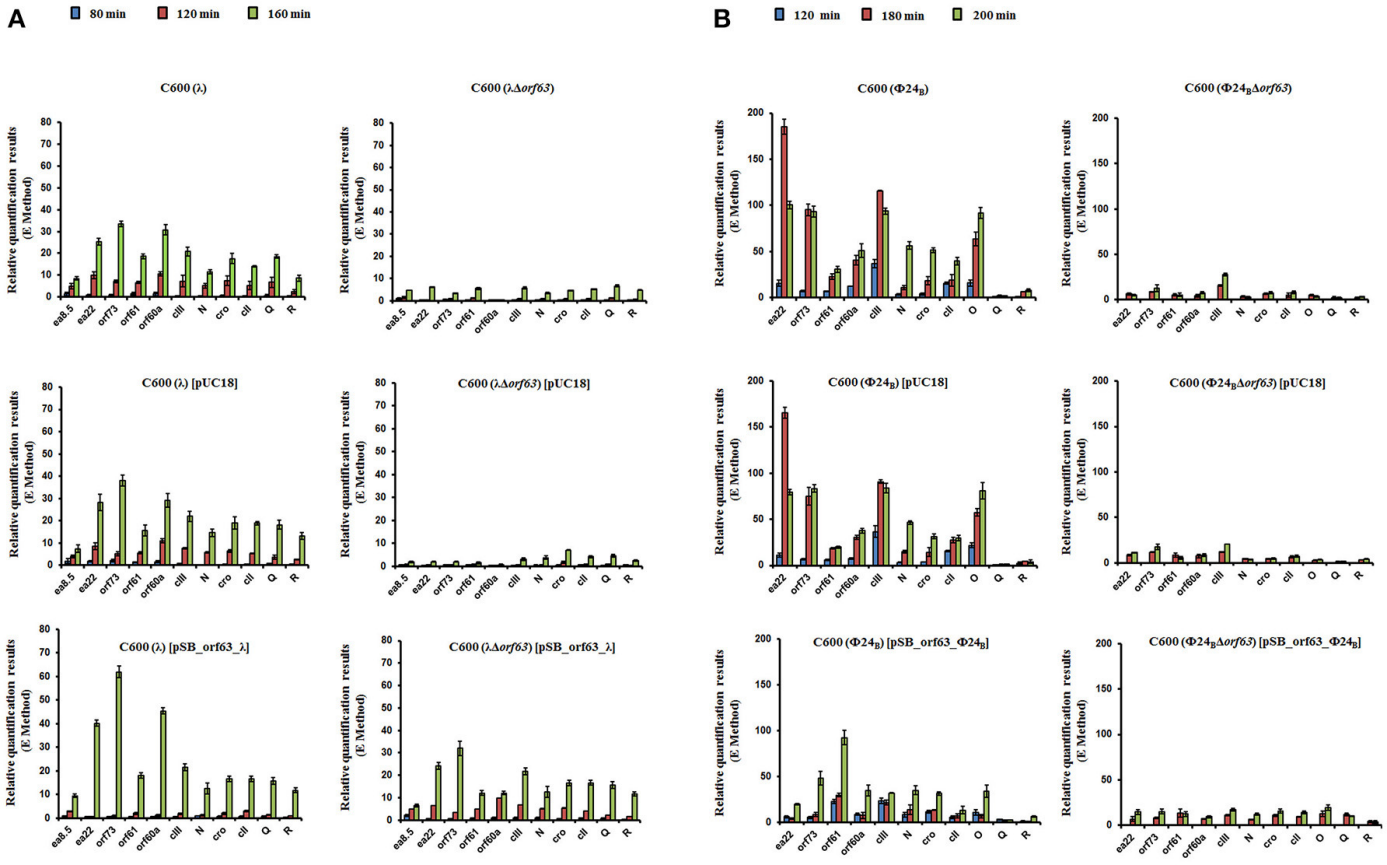
Levels of transcripts of indicated genes of wild type phages λ and Φ24_B_ and their deletion mutants in the absence or presence of control plasmid [pUC18] or plasmid bearing the deleted *orf63* [pSB_orf63], assessed by quantitative reverse RT-PCR analysis, aftser prophage induction with 1 mM H_2_O_2_ in *E. coli* C600 host at 30°C. Levels of transcripts corresponding to particular genes were determined at following times after induction: 80 (blue), 120 (red) or 160 (green) minutes in case of phages λ and λΔ*orf63* **(A)**, and 120 (blue), 180 (red) or 200 (green) minutes in case of phages Φ24_B_ and Φ24_B_Δ*orf63* **(B)**. Results are presented as mean values from three independent experiments with error bars indicating *SD*.

Since experiments described above indicated that *orf63* function is involved in the regulation of lysogenization and prophage induction in both λ and Φ24_B_, we aimed to measure expression of selected bacteriophage genes in *E. coli* cells after hydrogen peroxide-provoked prophage induction. Reverse transcription quantitative real time PCR (RT-qPCR) was used to assess abundance of particular transcripts. We have measured expression levels of genes from the *exo-xis* region (*ea8.5, ea22, orf73, orf61, orf60a*) and some key regulatory genes of λ and Φ24_B_, i.e., *N, cro, c*II, *Q, R*. We found that expression of all tested genes was significantly impaired in Δ*orf63* mutants of both λ and Φ24_B_ relative to wild-type phages at all tested times after prophage induction (Figures 7A,B, respectively). These results confirm that prophage induction is significantly impaired in the absence of *orf63*, and suggest a regulatory role for the *orf63* gene product in the control of expression of phage genes. In the case of phage λ, complementation of the Δ*orf63* mutation by overexpression of wild-type *orf63* from a plasmid was successful, at least at certain times after prophage induction (Figure 7A). However, we failed to obtain such a complementation in phage Φ24_B_ (Figure 7B). This might suggest that specific ratio(s) of Orf63 is/are required for accurate regulation of phage development.

## Discussion

Considering that the complete 48,502 bp genome of λ was achieved in 1983, it is astounding that this well-investigated model virus still contains uncharacterized open reading frames, many of which lie between the *exo*, and *xis* genes. We began this investigation by demonstrating that *orf63* found within the *exo-xis* region, encodes a *bona fide* protein both in structural, and functional terms. Consistent with its name, this small 63 aa. protein is comprised of only two helices that cover most of the available sequence. Analytical gel filtration of purified Orf63 suggests that the oligomeric state is a trimer. If Orf63 deviates significantly from a globular shape, it is possible that the molecular weight may be overestimated by the gel filtration assay, the oligomeric state could be a dimer. However, given the significant line broadening observed during a series of initial NMR based surveys performed at 25°C that could only be alleviated by performing all of the studies subsequently at 37°C, the NMR data tend to corroborate the gel filtration findings.

Since Orf63 appears to be a functional protein, we tested effects of deletion of *orf63* on development of bacteriophages λ and Φ24_B_. In the absence of functional Orf63, we observed a significant increase in the efficiency of lysogenization, and considerable lower efficiency of hydrogen peroxide-mediated prophage induction. These results may suggest that Orf63 is involved in the regulation of expression of specific phage genes. Studies with the use of RT-qPCR revealed that expression of vast majority of crucial regulatory genes, as well as genes from the *exo-xis* region, is significantly influenced by the absence of *orf63*. Moreover, perhaps specific ratio of Orf63 to other regulators is required, as it was impossible to obtain complementation with the wild-type *orf63* expressed from a plasmid in Φ24_B_, though it was successful in λ. The hypothesis about the requirement of specific Orf63 ratio(s) for accurate regulation of phage development is supported by impaired expression of Φ24_B_ genes during overexpression of *orf63*.

**Figure 8:**
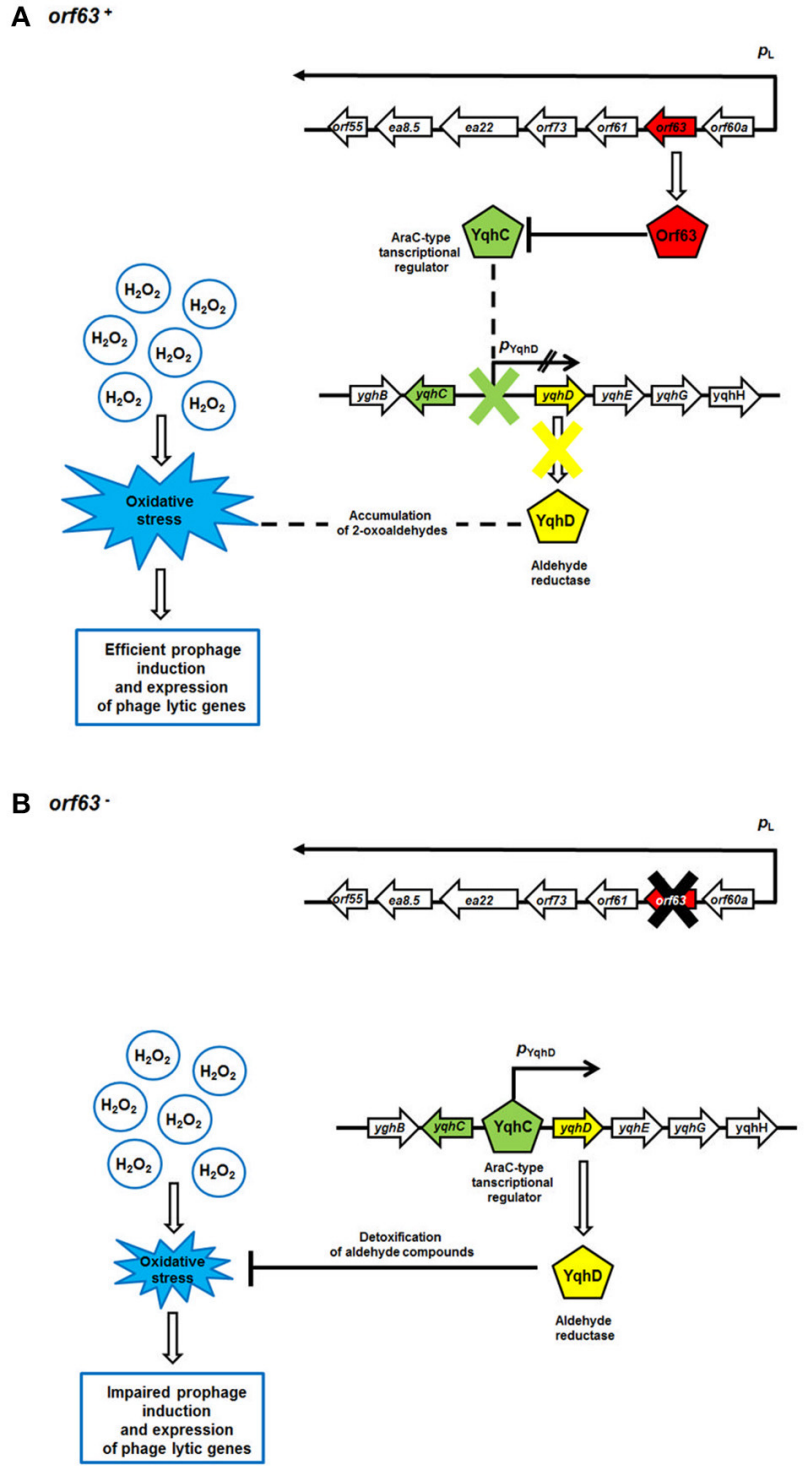
A model for Orf63-mediated modulation of bacteriophage development. Mechanisms operating in the presence **(A)** or absence **(B)** of the orf63 function are shown. Transcripts are shown as black arrows, with arrowheads indicating directionality of transcription. The lack of the activity of the *p*_YghD_ promoter is marked as two black inclined lines. Phage and *E. coli* (host) proteins are presented as pentagons. Stimulations of processes or protein production/activity are shown by open arrows. Negative regulations of processes or protein functions are represented by blunt-ended lines.

A previous yeast two-hybrid study identified the transcription factor YqhC as a possible protein partner of Orf63 (Blasche et al., 2013). YqhC is interesting because it is a transcription factor that promotes the synthesis of YqhD, the major enzyme responsible for detoxifying compounds produced from glucose under conditions of oxidative stress (Lee et al., 2010). Among the compounds that YqhD acts upon are 2-oxoaldehydes, toxic and highly reactive products of oxidative stress on the bacterium formed from glucose. It has been proposed that oxoaldehydes are one class of compound that is capable of inducing a wider stress response through SoxRS (Benov and Fridovich, 2002). Since neutrophils mount a vigorous oxidative attack during a STEC infection, Orf63 may be beneficial to the bacteriophage by manipulating the microenvironment of the bacterial host. Orf63 by binding the transcriptional activator YqhC, may prevent it from attenuating the stress response created by neutrophil mediated attack on EHEC strains in the gut, and promoting a transition to the lytic phase commensurate with the activation of associated phage genes in that response. A putative mechanism for Orf63-mediated modulation of prophage induction and phage genes' expression is presented in Figure 8. Notwithstanding its role in protein-protein interactions, it is still possible that Orf63 itself could function as a transcriptional regulator, although its small size, lack of a known DNA binding domain, and relatively few basic amino acids argue against this possibility. A high-resolution structure of Orf63 alone, or in complex with a possible interactor like YqhC, will resolve these outstanding questions.

In conclusion, Orf63 is a folded protein and has structural properties suggesting its regulatory role. Analyses of mutants of bacteriophages λ and Φ24_B_ devoid of *orf63* indicated its function in the control of bacteriophage development at the stages of lysis vs. lysogenization decision and prophage induction. Further studies will include determination of biochemical properties of Orf63 and its possible interactions with phage- and/or host-encoded proteins, as well as with phage DNA, to understand molecular mechanisms of its function as a modulator of phage development.

## Author contributions

AD and SB contributed equally to this work. AD, SB, BN, GT, TG, and AN took part in the physiological studies on bacteria and phages and data processing. AD, SB, and BN participated also in writing the manuscript and planning of the study. AR, SP, and LD performed the protein analyses. GW, LD, and AW were engaged in writing the manuscript and took part in planning of the study and discussions.

### Conflict of interest statement

The authors declare that the research was conducted in the absence of any commercial or financial relationships that could be construed as a potential conflict of interest. The reviewer RO and handling Editor declared their shared affiliation, and the handling Editor states that the process nevertheless met the standards of a fair and objective review.
